# Comparison of epitheliotrophic factors in platelet-rich plasma versus autologous serum and their treatment efficacy in dry eye disease

**DOI:** 10.1038/s41598-022-12879-x

**Published:** 2022-05-26

**Authors:** Chanatip Metheetrairut, Panotsom Ngowyutagon, Abhirak Tunganuntarat, Ladawan Khowawisetsut, Kulvara Kittisares, Pinnita Prabhasawat

**Affiliations:** 1 Department of Biochemistry, Faculty of Medicine Siriraj Hospital, Mahidol University, Bangkok, Thailand; 2 Department of Ophthalmology, Faculty of Medicine Siriraj Hospital, Mahidol University, 2 Wanglang Road, Bangkok Noi, Bangkok, 10700 Thailand; 3 Department of Parasitology, Faculty of Medicine Siriraj Hospital, Mahidol University, Bangkok, Thailand; 4 Department of Transfusion Medicine, Faculty of Medicine Siriraj Hospital, Mahidol University, Bangkok, Thailand

**Keywords:** Biochemistry, Diseases, Medical research

## Abstract

Current treatment of severe dry eye disease (DED) includes blood-derived eye drops, such as autologous serum (AS), which lubricate the eyes and provide factors that improve ocular surface and aid in wound healing. Recent studies indicated that platelet-rich plasma (PRP) was also effective. This study aims to compare the concentration and stability of epitheliotrophic factors in AS and PRP and their efficacy in DED patients. Epitheliotrophic factors of interest are epidermal growth factor (EGF), fibronectin, platelet-derived growth factor-AB (PDGF-AB), and transforming growth factor-beta1 (TGF-β1). We determined that all epitheliotrophic factors were present in AS and PRP at baseline and did not decrease in concentrations in all storage conditions (4 °C for 1 week and at − 20 °C for 1 and 3 months). However, differences in concentrations in AS and PRP were observed. PRP was also shown not to be inferior to AS in terms of efficacy in DED treatment in a prospective randomized control trial which evaluated ocular surface disease index, dry eye questionnaire, ocular surface staining, tear breakup time, and Schirmer test at baseline and at 1-month follow-up. Therefore, with its shorter preparation time, PRP could be considered as an alternative to AS for the treatment of DED.

## Introduction

Dry eye disease (DED) is one of the most commonly found eye diseases with the prevalence ranging from 8.7 to 30.1%^1^. DED has a negative impact on both physiological and psychosocial aspects of patients^2^. The initial management of DED usually includes artificial tear replacement. However, for severe DED, blood-derived eye drops such as autologous serum and platelet-derived eye drop have been recommended as one of the choices in step-3 DED management algorithm according to Dry Eye Workshop II (DEWS II)^3^. That is because while both artificial tear and blood-derived eye drop provide lubrication for dry eyes, blood-derived eye drops have additional factors that can improve ocular surface and facilitate wound healing. Previous reports demonstrated that 20% autologous serum is effective in treating severe dry eye and is used regularly in clinical setting^4–6^. Recently, platelet-derived eye drop has also been shown to produce a significant improvement or disappearance of DED symptoms^7–10^. The superiority of autologous serum and platelet-derived eye drop over artificial tear is considered to be from the presence of epitheliotrophic factors in autologous serum and platelet-derived eye drop, which is similar to those presented in natural tear. Important epitheliotrophic factors are epidermal growth factor (EGF), transforming growth factor-beta1 (TGF-β1), fibronectin, and platelet-derived growth factor-AB (PDGF-AB)^11^. These epitheliotrophic factors play an important role in several processes, including cell migration, cell proliferation, wound healing, production of important extracellular matrices, and chemotactic effects^12–19^.

There are a few studies comparing the concentration of epitheliotrophic factors in autologous serum and different types of platelet-derived eye drop, including platelet-rich plasma, plasma rich in growth factors, platelet lysate (depending on preparation protocol). These studies show that platelet-derived eye drop has a higher concentration of epitheliotrophic factors than autologous serum does^20,21^. However, differences in preparation techniques might also affect the concentration of epitheliotrophic factors^20–25^. Stability of the epitheliotrophic factors in autologous serum and platelet-rich plasma over a long period of storage time is also one of the major concerns regarding their uses. Phasukkijwatana et al.^24^ reported that epitheliotrophic factors in autologous serum were stable for up to 6 months, if stored properly at − 20 °C. Another study focused on the stability of plasma rich in growth factors (PRGF) eye drops and demonstrated that PDGF-AB, VEGF, EGF, and vitamin A stored at − 20 °C for 15, 30, and 90 days were stable, but fibronectin and TGF-β1 stored in the same condition were not^22^. However, to the best of our knowledge, no study on the comparison of factors in platelet-rich plasma and autologous serum storing in various conditions in patients with DED has been done so far.

In addition to the difference in concentration of epitheliotrophic factors and their stability, it is also crucial to examine the difference in clinical outcomes. There has been no study that compared between the efficacy of platelet-rich plasma and autologous serum in DED before.

The aim of this study was to evaluate the concentration and stability of epitheliotropic factors in 20% platelet-rich plasma (PRP) and 20% autologous serum (AS) to compare both types of blood-derived eye drop at similar dilution. Additionally, we conducted a prospective double-masked randomized control trial to compare the clinical efficacy of PRP and AS in DED.

## Methods

### Subjects

This study followed the tenets of the Declaration of Helsinki and was approved by Siriraj IRB protocol number 013/2562(EC4). Informed consent was obtained from all subjects after an explanation of the study and its possible consequences. This trial was prospectively registered on Thai clinical trial registry on 13/11/2019 (study ID: TCTR20191119001). All recorded data were carried out in the Department of Ophthalmology, Faculty of Medicine Siriraj Hospital, Mahidol University, Thailand between November 2019 and February 2020.

A total of 10 patients with dry eye disease were enrolled in this study. All patients met the following criteria: (1) dry eye symptoms (Ocular Surface Disease Index (OSDI ≥ 13)) and (2) one of these signs; fluorescein tear breakup time (FTBUT) < 10 s; abnormal ocular surface staining (> 5 corneal spots, or > 9 conjunctival spots). Exclusion criteria were age under 18 years old, use of PRP or AS within 2 weeks before enrollment, use of immunosuppressive drug, blood transmitted diseases, pregnancy, systemic underlying diseases such as uncontrolled diabetes mellitus, chronic kidney disease, liver cirrhosis, or systemic infection, and unequal severity of dry eye disease between both eyes.

### Preparation of PRP and AS

Peripheral blood was collected from subjects for both PRP and AS. For PRP preparation, 35 ml of peripheral blood was collected into a 50-ml tube with 3.8% sodium citrate as anticoagulant (final concentration of 0.475% sodium citrate) and centrifuged at 2200×*g* for 10 min at 22 ± 2 °C. Plasma was then collected. For AS preparation, 35 ml of peripheral blood was also collected into a 50-ml tube and incubated at 31 °C for 2 h to allow to clot and then centrifuged at 3000×*g* for 30 min at 4 °C. The supernatant serum was collected. Collected plasma and serum were diluted from 100 to 20% with balanced salt solution (BSS). An aliquot of each sample was kept for quantification of growth factors and fibronectin, while the rest of the samples were aliquoted into 5-ml amber bottles and prescribed to patients. All procedures were performed under sterile conditions. The patients were instructed to keep the prepared PRP and AS bottles frozen, while storing the bottle in use at 4 °C.

### Measurement of the concentration of epitheliotrophic factors

The concentrations of EGF, TGF-β1, fibronectin, and PDGF-AB in PRP and AS samples were quantified by enzyme-linked immunosorbent assay (ELISA) kits according to the manufacturers’ instruction (R&D Systems, Minneapolis, MN, USA).

**Figure 1:**
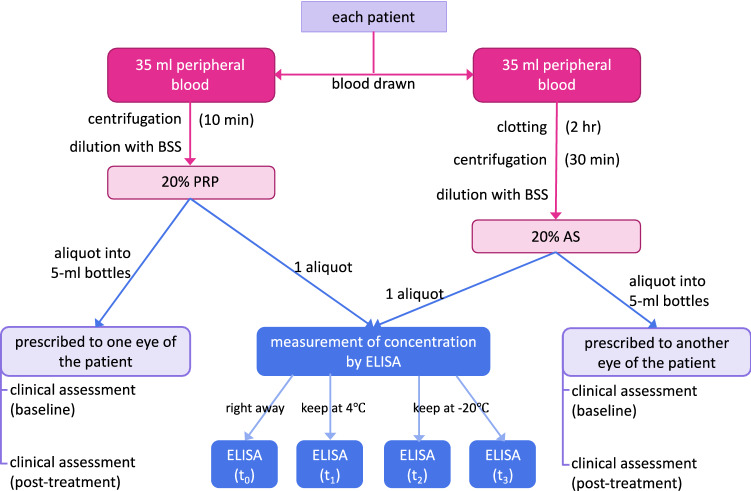
A summary of the design of the study. The diagram shows the process done for each patient; these processes were performed for all 10 patients in parallel. *PRP* platelet-rich plasma, *AS* autologous serum, *ELISA* enzyme-linked immunosorbent assay. The details of the methods are stated in the “Methods” section.

Fresh samples were assayed immediately for baseline (t_0_) epitheliotrophic factor concentrations. Samples were aliquoted and stored at 4 °C or − 20 °C for a specific duration before being assayed: 4 °C for 1 week (t_1_), − 20 °C for 1 month (t_2_) and − 20 °C for 3 months (t_3_) (Fig. 1).

### Clinical assessment

Each subject was randomly assigned PRP to one eye and AS to the other eye every hour for 1 month using simple randomization. Subjects were not informed which eye received PRP or AS. The physical properties of PRP and AS were similar. The bottles containing PRP and AS were also similar in appearance except for the cap color and the instruction label indicating which eye to administer its content to prevent confusion.

Dry eye symptoms were evaluated with OSDI and dry eye questionnaire, modified from The Osaka Study^26^. Signs of dry eye were evaluated with conjunctival and corneal staining according to Oxford Staining Score^27^, fluorescein tear breakup time, and Schirmer test without anesthetic. Best-corrected visual acuity (BCVA) and intraocular pressure (IOP) were measured for safety evaluation at baseline and 1 month by the same masked assessor. Moreover, the patients were asked about adverse events and their preference for PRP or AS during their 1-month visit (Fig. 1).

### Statistical analysis

The primary objective of this study was to compare the concentration of the epitheliotrophic factors, EGF, TGF-β1, fibronectin, and PDGF-AB, in PRP and AS. According to Anitua et al.^20^ the PDGF-AB concentration was 14.1 ± 4.9 ng/ml in PRGF and 3.3 ± 0.1 ng/ml in AS. In order to achieve a probability value (*p*-value) with type I error of 0.01, 2-sided and a 95% power with type II error of 0.05, the minimal requirement was 8 subjects, as calculated with nQuery Advisor. For the primary outcome, the differences in concentration of EGF, TGF-β1, fibronectin, and PDGF-AB between in PRP and AS were analyzed by Wilcoxon signed rank test. The difference of concentration of EGF, TGF-β1, fibronectin, and PDGF-AB in PRP and AS in the different storage condition and time were used for the stability analysis, which was assessed by Friedman’s two-way analysis of variance. The differences in factor stability were analyzed using Dunn’s test, with a level of significance set at p < 0.05. The changes of clinical parameters from baseline were obtained at 1 month and used for the efficacy and comparative efficacy analysis with Wilcoxon signed rank test. Statistical analyses were performed with PASW statistics (SPSS software version 18.0; SPSS Inc, Chicago IL).

## Results

**Figure 2:**
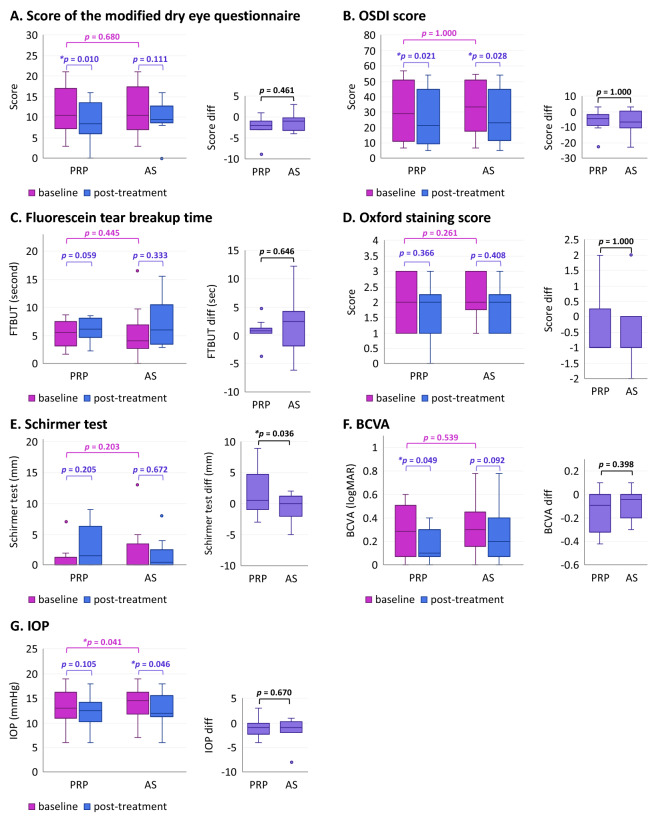
Characteristics of the patients at baseline and clinical outcomes. The patients were evaluated for their (**A**) score of the modified dry eye questionnaire, (**B**) OSDI score, (**C**) Fluorescein tear breakup time (second), (**D**) Oxford staining score, (**E**) Schirmer test (mm), (**F**) BCVA (LogMAR), and (**G**) IOP (mmHg). Median and interquartile range are shown in box plots (N = 10 for each group). The left panels of (**A–G**) show the values at baseline and clinical outcomes after treatment with PRP or AS; while the right panels of (**A–G**) show within-subject differences in scores or measurements after treatment. *p-values designate those that are statistically significant; *p*-values (without *) designate those that are not statistically significant, as analyzed by Wilcoxon signed rank test.

A total of 20 eyes of 10 patients were included in this study. All patients were female with mean age of 65.6 ± 14.35 years. There was no statistically significant difference in baseline BCVA, score of the modified dry eye questionnaire, OSDI, FTBUT, Oxford staining score, and Schirmer test without anesthetic between both eyes (Table 1, Fig. 2A–F).

**Table 1:** Characteristics of the patients at baseline.

	PRP (N = 10)	AS (N = 10)	*p-*value (N = 10)
Score of the modified dry eye questionnaire	10.5 (3–21)	10.5(3–21)	0.680
OSDI score	29.065 (6.82–56.81)	33.335 (6.82–54.55)	1.000
Fluorescein tear breakup time (second)	5.535 (1.75–8.76)	4.135 (0–16.51)	0.445
Oxford staining score	2 (1–3)	2 (1–3)	0.261
Schirmer test (mm)	0 (0–7)	0 (0–13)	0.203
BCVA (LogMAR)	0.29 (0–0.6)	0.3 (0–0.78)	0.539
IOP (mmHg)	13 (6–19)	14.5 (7–19)	**0.041**

All data presented as median (range) and analyzed by Wilcoxon signed rank test.

*p-*values in bold are statistically significant.

### Comparison of the concentrations of the epitheliotrophic factors in AS and in PRP

**Figure 3:**
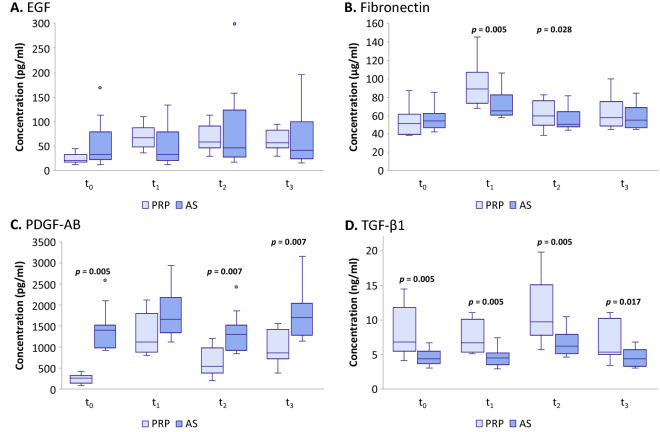
Concentrations of epitheliotrophic growth factors and fibronectin in 20% platelet-rich plasma (PRP) and 20% autologous serum (AS) at baseline (t_0_), after storage at 4 °C for 1 week (t_1_), and after storage at − 20 °C for 1 month (t_2_) and 3 months (t_3_). Median and interquartile range of concentrations are shown in box plots (N = 10 for each group). The presence of *p-*value above any pair of data signifies that the difference between concentrations in AS and those in PRP in that condition is statistically significant as analyzed by Wilcoxon signed rank test. Conversely, the absence of *p*-value above any pair of data signifies no statistically significant difference for that comparison. The factors shown here are: (**A**) EGF, (**B**) fibronectin, (**C**) PDGF-AB, and (**D**) TGF-β1.

We measured the concentrations of 4 epitheliotrophic factors, EGF, fibronectin, PDGF-AB, and TGF-β1, and compared between those in AS and those in PRP. We have demonstrated that the concentrations of EGF, fibronectin, and TGF-β1 were higher in PRP than in AS at two or more time points in both storage temperature, although only the differences in concentration of fibronectin and TGF-β1 reached statistical significance (*p*-values of 0.005 and 0.028 for t_1_ and t_2_ of fibronectin and *p*-values of 0.005, 0.005, 0.005, and 0.017 for t_0_, t_1_, t_2_, and t_3_ for TGF-β1) (Fig. 3A,B,D). On the other hand, the concentrations of PDGF-AB were significantly higher in AS than in PRP at baseline (*p*-value of 0.005) and after being stored at − 20 °C (*p*-values of 0.007 and 0.007 for t_2_ and t_3_) (Fig. 3C).

### Concentrations of the epitheliotrophic factors in PRP and AS after storing in various conditions

Next, we examined how the concentrations of epitheliotrophic factors were affected by storage conditions. We advised patients to store unopened AS or PRP bottles in the freezer section of household refrigerators, which we approximated by storing at − 20 °C, and the bottles in use in the cooling section of household refrigerators, which we approximated to be at 4 °C. If the patients used AS or PRP hourly as prescribed, we estimated that one bottle should last one week at most. Therefore, we examined AS and PRP after storing at 4 °C for 1 week (t_1_) and at − 20 °C for 1 month (t_2_) and 3 months (t_3_), and then compared them to the measurement at baseline (t_0_).

**Figure 4:**
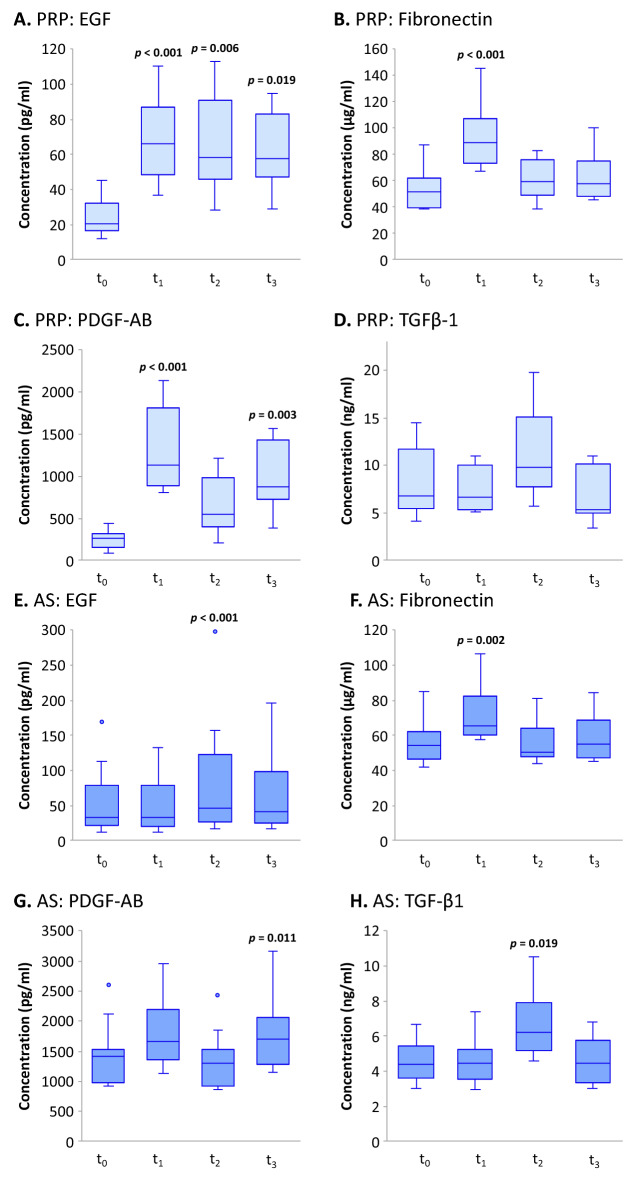
Concentrations of epitheliotrophic growth factors and fibronectin at baseline (t_0_), after storage at 4 °C for 1 week (t_1_), and after storage at − 20 °C for 1 month (t_2_) and 3 months (t_3_). Median and interquartile range of concentrations are shown in box plots (N = 10 for each group). The presence of *p-*value above data at any conditions signifies that the difference between concentrations at baseline (t_0_) and those at that condition (t_1_, t_2_, or t_3_) is statistically significant as analyzed by Dunn’s test. Conversely, the absence of *p*-value above data at any conditions signifies no statistically significant differences between those at baseline (t_0_) and at that condition (t_1_, t_2_, or t_3_). The factors shown here are: (**A,E**) EGF; (**B,F**) fibronectin; (**C,G**) PDGF-AB; and (**D,H**) TGF-β1; when (**A–D**) are concentrations in platelet-rich plasma (PRP) and (**E–H**) are those in autologous serum (AS).

After storage at 4 °C for 1 week, the concentrations of EGF, fibronectin, and PDGF-AB in PRP significantly increased from those at baseline (*p*-values < 0.001 for all three factors); however, this phenomenon was not observed for the concentration of TGF-β1 in PRP stored in the same condition (Fig. 4A–D). Furthermore, after being stored at − 20 °C, only the concentrations of EGF and PDGF-AB in PRP were significantly higher than that at baseline at at least one time point (*p*-values of 0.006 and 0.019 for 1-month and 3-month time points of EGF and *p*-value of 0.003 for t_3_ of PDGF-AB) (Fig. 4A,C). The concentrations of fibronectin and TGF-β1 in PRP in storage at − 20 °C were unchanged from those at baseline at both time points (Fig. 4B,D).

In contrast, the concentrations of EGF, PDGF-AB and TGF-β1 in AS were not significantly different when they were stored at 4 °C from that at baseline, but the concentration of fibronectin slightly increased from baseline concentration (*p-*value of 0.002) (Fig. 4E–H). Similarly, after storing at − 20 °C, the concentrations of EGF, TGF-β1, and PDGF-AB in AS were increased slightly after one duration of storage but not the other: 1 month for EGF and TGF-β1 (*p*-values of < 0.001 and 0.019 respectively) (Fig. 4E,H) and 3 months for PDGF-AB (*p*-value of 0.011) (Fig. 4G). However, the concentration of fibronectin in AS was not different from that at baseline of AS after being stored at − 20 °C for any lengths of time (Fig. 4F).

### Comparison of the efficacy of PRP and AS in DED

All 10 patients completed the trial period. The average number of drops was 10 times per day in each eye. There was no adverse events reported after using PRP or AS. No preference between PRP and AS was reported by the patients.

We examined the efficacy of PRP and AS in improving symptoms of DED by comparing the modified dry eye questionnaire score and OSDI score at baseline and post-treatment (Fig. 2A,B). We found that usage of PRP eye drops for 1 month significantly improved both the modified dry eye questionnaire and OSDI scores (Fig. 2A,B). On the other hand, usage of AS eye drops for 1 month appeared to improve both the modified dry eye questionnaire and OSDI scores; however, only the improvement in OSDI score reached statistical significance (Fig. 2A,B). When the post-treatment improvement was compared between those receiving PRP and AS to determine whether one has better efficacy, it was found that the post-treatment improvement in the dry eye questionnaire and OSDI scores were not significantly different between the two groups (Fig. 2A,B, right panels).

We further examined the signs of DED by FTBUT, Schirmer test, and Oxford staining score at baseline and post-treatment to evaluate the efficacy of PRP and AS (Fig. 2C–E). We determined that FTBUT, Schirmer test and Oxford staining score were slightly improved, though not significantly, after the use of PRP and AS (Fig. 2C–E). However, when the changes observed in Schirmer test was compared between the two groups, the differences were significantly higher for those receiving PRP than those receiving AS (Fig. 2C–E). Additionally, PRP eye drop significantly improved BCVA after 1 month of treatment (Fig. 2F).

## Discussion

This study demonstrated that PRP and AS both contained epitheliotrophic factors which are known to play important roles in cell migration, cell proliferation, and wound healing. Furthermore, this study showed that the three growth factors and fibronectin in PRP and AS were stable after storage at − 20 °C for at least 3 months. Moreover, we determined that PRP and AS were safe and effective in the treatment of DED.

In this study, we determined that all four epitheliotrophic factors of interest: EGF, fibronectin, PDGF-AB, and TGF-β1, were present in both PRP and AS eye drops as expected. However, there were some differences in concentration when compared between those in PRP and in AS (Fig. 3). Particularly, the concentrations of PDGF-AB was significantly higher in AS at baseline and after 2 storage conditions (Fig. 3C), while TGF-β1 was present at higher concentration in PRP at all time points (Fig. 3D). Fibronectin was present at similar concentration in both eye-drops at baseline but increased to be significantly higher in PRP after 2 storage conditions (Fig. 3B).

Furthermore, we ascertained that the concentrations of all four epitheliotrophic factors in both AS and PRP did not drop within the 3-month time frame that patients normally kept these blood-derived eye drops (Fig. 4). This result is consistent with previous studies which showed that EGF and fibronectin were stable in PRP and AS at − 20 °C for 3 months^24,28^. However, one study reported contrasting findings, in which they found that after storage at − 20 °C for up to 3 months, only EGF was stable in PRGF but fibronectin was not^29^. This might suggest that different preparations of blood-derived eye drop is the cause of the differences in stability and might indicate the superiority of PRP over PRGF. Our results confirm that both PRP and AS can be frozen for up to 3 months without a decrease in the concentrations of epitheliotrophic factors.

Surprisingly, when stored at 4 °C, EGF, fibronectin, and PDGF-AB in PRP increased from baseline concentrations and fibronectin also increased in AS. Particularly, EGF in PRP increased approximately 3 times from baseline to the concentration which exceeded that in AS (Figs. 3A, 4A,E). Additionally, we observed that EGF in PRP increased almost 3 times from baseline after storing at − 20 °C as well (Fig. 4A). Increase of certain epitheliotrophic factors may be explained by the activation of platelets in PRP, which is composed of blood plasma enriched with platelets. Platelet activation can occur in some conditions, such as storage duration, temperature, calcium chloride addition, etc.^28,30,31^. Previous studies and our results suggest that it could be beneficial for patients to use PRP after it has been frozen or refrigerated rather than the fresh preparation.

Many factors contribute to the different amount of epitheliotrophic factors in each subject; for example, age, gender, underlying diseases, drug use, and daily activity and routine^23,32,33^. The difference in PRP and AS preparation methods also contribute to the differences in epitheliotrophic factor concentrations. That may explain why our results of concentration of epitheliotrophic factors were different from those in some previous studies^28^. When comparing with the study in human tear, we found that TGF-β1 and fibronectin concentrations in PRP were similar to those in human tear but EGF concentration in PRP was lower^13^. However, our results on clinical efficacy suggest that despite some variations in factor concentrations, PRP is likely to have enough growth factors and fibronectin to be useful in treating DED patients.

This study showed the efficacy of PRP and AS in DED treatment based on improvement of symptoms and sign of DED (Fig. 2). PRP might be superior to AS due to its ability to significantly improve dry eye questionnaire score, OSDI score, and BCVA, whereas the improvement of only OSDI score was observed in AS-treated group when compared to baseline (Fig. 2A,B,F). For Schirmer’s test, while the improvement from treatment over baseline scores did not reach significant levels in either group, it was observed that PRP-treated eyes had significantly more improvement than AS-treated eyes when comparing between groups (Fig. 2E). These results were of similar patterns to those reported in previous studies that investigated the use of AS or PRP compared to artificial tears in DED^6,8,34^. Another factor that PRP may be preferred over AS is that the preparation procedure of AS includes an additional 2-h incubation period. In clinical practice, this additional waiting time could create inconvenience for patients and influence their preference.

The strength of this study was that it measured the concentrations of epitheliotrophic factors and their stability in the same samples prescribed to patients and analyzed for clinical efficacy. Moreover, since the clinical study design included the use of PRP and AS in each eye of the same patients, epitheliotrophic factor concentrations were also measured in PRP and AS of the same persons to lessen variability due to lifestyle and individual’s health. Additionally, the samples were derived from dry eye patients rather than those of healthy volunteers which might be different due to demographic and underlying diseases.

However, the disadvantage of this study was that all recruited subjects were female, which may be due to the higher prevalence of DED in female^1^. Therefore, the concentrations of epitheliotrophic growth factors in this study may be influenced by gender. The number of participants in this study was limited because we would like to test the same samples in the laboratory and clinical study. Future study comparing the clinical efficacy between PRP and AS may aim to recruit more patients, especially some of male gender. Additionally, it would be interesting to study which storage condition releases the most epitheliotrophic factors and the optimal concentration of epitheliotrophic factors in wound healing of ocular surface.

In conclusion, PRP could be considered as an alternative to AS for the treatment of DED because its efficacy and the concentration of epitheliotrophic factors presented were not inferior to those of AS, while PRP requiring much shorter preparation time. Moreover, this study also shows that PRP eye drops can be stored at − 20 °C for up to at least 3 months without decrements of epitheliotrophic factors concentration.

## Data Availability

All data generated or analyzed during this study are included in this published article.
